# Sperm DNA Fragmentation in Male Infertility: Tests, Mechanisms, Meaning and Sperm Population to Be Tested

**DOI:** 10.3390/jcm13175309

**Published:** 2024-09-07

**Authors:** Donata Conti, Costanza Calamai, Monica Muratori

**Affiliations:** Department of Experimental and Clinical Biomedical Sciences “Mario Serio”, University of Florence, 50139 Florence, Italy; donataconti12@gmail.com (D.C.); costanza.calamai@unifi.it (C.C.)

**Keywords:** sperm DNA fragmentation, TUNEL, SCSA, COMET Assay, SCD test, tip of iceberg theory, abortive apoptosis, defect in chromatin maturation, oxidative stress, assisted reproductive technologies

## Abstract

Sperm DNA fragmentation (sDF) is a DNA damage able to predict natural conception. Thus, many laboratories added tests for the detection of sDF as an adjunct to routine semen analysis with specific indications. However, some points related to sDF are still open. The available tests are very different each from other, and a direct comparison, in terms of the prediction of reproductive outcomes, is mandatory. The proposed mechanisms responsible for sDF generation have not yielded treatments for men with high levels of sDF that have gained the general consent in clinical practice, thus requiring further research. Another relevant point is the biological meaning to attribute to sDF and, thus, what we can expect from tests detecting sDF for the diagnosis of male infertility. SDF can represent the “tip of iceberg” of a more extended and undetected sperm abnormality somehow impacting upon reproduction. Investigating the nature of such a sperm abnormality might provide novel insights into the link between sDF and reproduction. Finally, several studies reported an impact of native sDF on assisted reproduction technique outcomes. However, to fertilise the oocyte, selected spermatozoa are used where sDF, if present, associates with highly motile spermatozoa, which is the opposite situation to native semen, where most sDF associates with non-viable spermatozoa. Studies comparing the impact of sDF, as assessed in both native and selected spermatozoa, are needed.

## 1. Introduction

Infertility affects 48 million couples [1] worldwide, and the male factor accounts for approximately 50% of these cases, with it being a primary or contributing factor. Mostly, male infertility is due to an impaired spermatogenesis [2], although the causes of such impairment are unknown in a large percentage of men. Spermatogenesis is a complex process stemming from spermatogonia and producing, through meiosis, haploid round spermatids that proceed into spermiogenesis to become spermatozoa. Further, after releasing from the seminiferous epithelium and during the transit in the epididymis, spermatozoa undergo additional modifications, including the acquisition of motility and the completion of chromatin compaction [3,4].

The quality of the spermatogenetic process is assessed by routine semen analysis, determining the main sperm parameters, namely the concentration, number, motility, and morphology of spermatozoa. Although this test is a cornerstone in the diagnosis of male infertility, its ability to predict natural conception is quite limited [5,6]. Indeed, failure to accomplish the task may depend on defects in the sperm structure/function that are subtler than those detected by routine semen analysis. In addition, ejaculated spermatozoa have to acquire crucial functions in the female genital tract like capacitation, in turn priming male gametes to trigger acrosome reaction, acquiring a particular motility pattern known as hyperactivation and binding the oocyte. Finally, an integer paternal genome has to be delivered to the oocyte, along with the proper epigenetic cargo made of DNA methylation patterns, non-coding RNAs, protamines, and histones, which are crucial for fertilization and early embryo development [7]. This picture, limited by the current knowledge, indicates that spermatozoa have to simultaneously accomplish many requirements in order to be able to fertilise the oocyte and successfully support the next steps of embryo development. Hence, it is unlikely that testing a single parameter is sufficient to evaluate the fecundating potential of an ejaculate. On the other hand, a relevant sperm defect impairing one of the many functional requirements has to be extended to the entire cell population in order to impede reproduction. Another important point is the sperm population which should be tested. In natural reproduction, due to the drastic selection during the transit in the female genital tract [8], only a small sperm population will reach the oocyte with competence for fertilization, and, in principle, such a population appears to be the most suitable target for diagnostic tests.

In this complex scenario, it is quite surprising that the detection of just one parameter, namely sperm DNA fragmentation (sDF), in the bulk of native semen samples, is able to discriminate between fertile and infertile subjects, as indicated by several reports [9,10,11,12] (see below for further discussion on this point).

### SDF and Its Use as an Adjunct of Routine Semen Analysis

SDF is a DNA damage consisting of the presence of both single- and double-stranded DNA breaks, and its amounts correlate to, but are not completely dependent upon, poor semen quality [9]. At variance with routine semen parameters [5,6], sDF is able to predict natural conception [9,10], irrespective of the test used to detect it [13], and when its level is over a proper threshold, the probability of achieving a natural pregnancy is drastically reduced [14]. SDF has been extensively investigated in the last decades, and currently, many laboratories add the analysis of sDF to routine semen analysis in the work-up of male infertility for specific patients [15,16]. In particular, sDF testing has been suggested for men with unexplained infertility or recurrent pregnancy loss or failure in ARTs [17,18]. Among the latter, patients with varicocele, irrespective of semen quality, have also been indicated as suitable for sDF determination [17].

However, a debate is still ongoing about the use of sDF determination in clinical practice, and several points related to sDF are still open. This review will discuss some of these points, including a comparison between the available tests for sDF detection, the mechanisms inducing sDF that are related to the strategies to treat men with high levels of sDF, the meaning to attribute to sDF, and the sperm population where sDF should be detected.

## 2. Tests for sDF Detection

There are four main tests for sDF detection used in clinical practice, namely SCSA (sperm chromatin structure assay), TUNEL (terminal transferase dUTP nick-end labelling), SCD (sperm chromatin dispersion) test, and alkaline comet assay (or single-cell gel electrophoresis). These tests are very different from each other in many aspects, as briefly described below and in Table 1. Among these tests, SCSA and TUNEL use flow cytometry, but TUNEL can use also fluorescence microscopy. Flow cytometry allows for a rapid assessment of thousands of spermatozoa per sample in an automatic manner, thus guaranteeing sound statistical results.

SCSA

**Figure 1:**
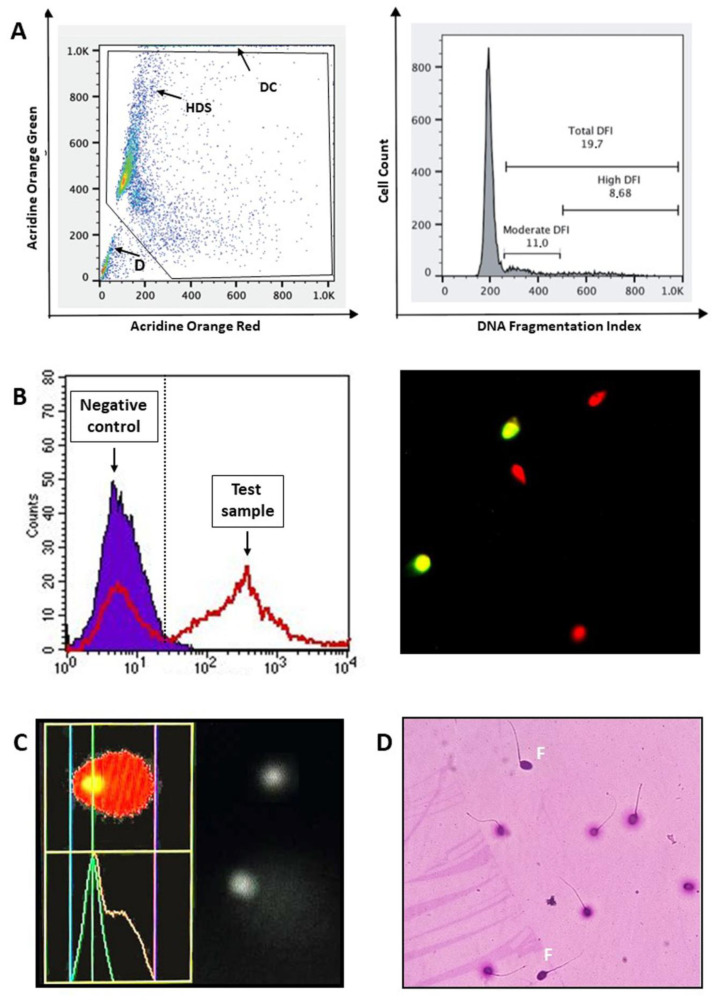
The main tests available for sDF detection. (**A**). SCSA. Left panel: AO-Green/AO-Red fluorescence dot plot. After excluding debris and diploid cells with a proper gate, the software calculates the DFI by the ratio red/(red + green) AO fluorescence from the raw data. DFI is represented as frequency histogram (right panel), where the percentages of DNA fragmented spermatozoa are determined (%DFI). DFI, DNA fragmentation index; DC, diploid cells; D, debris; HSD, high DNA stainability. (**B**). TUNEL. Left panel: Frequency histogram of TUNEL labelling. A negative control (absence of the enzyme TdT, solid histogram) is prepared for each patient in order to set a threshold beyond which spermatozoa are considered DNA fragmented in the test sample (open histogram). Right panel: Image of TUNEL labelling, as observed by fluorescence microscopy, showing spermatozoa with DNA fragmentation (green). Sample is counterstained by propidium iodide (red). (**C**). COMET assay. Typical patterns of spermatozoa with and without DNA fragmentation. In the former, the calculation of tail fluorescence intensity by software for image analysis is also shown. (**D**). SCD test. Typical patterns of spermatozoa with (without halo) and without DNA fragmentation (with halo). F, fragmented. The images of SCSA and COMET assay were kind gifts by, respectively, Dr. Giorgio Leter (ENEA Casaccia Research Center, Rome, Italy) and Prof. Lisa Giovannelli (Department NEUROFARBA, University of Florence, Florence, Italy).

This test stains spermatozoa with acridine orange (AO), after the induction of DNA denaturation by a slight acidification [19]. Under blue light (488 nm) excitation, AO fluoresces green when bound to double-stranded DNA and shifts to red when bound to single-stranded DNA (Figure 1A, left panel). Hence, for each cell, the ratio between red and total (green + red) AO fluorescence is computed and reported as a frequency histogram. Here, the percentages of DNA-fragmented spermatozoa (%DFI [20]) are determined as cells outside the main sperm population (Figure 1A, right panel). As described, this test does not directly detect the DNA breakage but, rather, the susceptibility of sperm chromatin to the induced denaturation. However, the more fragmented the DNA is, the more it is susceptible to denaturation. Hence, the SCSA results well correlate with tests directly detecting DNA breaks [21]. SCSA also detects sperm with high DNA stainability (HDS), which is interpreted as cells with intact DNA but immature chromatin [12] (Figure 1A, left panel).

TUNEL

The main characteristic of this test is the enzyme terminal deoxynucleotidyl transferase (TdT), which is a primer and template-independent DNA polymerase able to label both single- and double-stranded DNA fragments (blunt-ended or 5′-recessed DNA fragments) at the 3′-OH ends. The labelling is due to the incorporation of fluorescent nucleotides then revealed by fluorescence microscopy or flow cytometry (Figure 1B). Both of these types of instrumentation give results as percentages of DNA fragmented spermatozoa. However, the measures obtained by flow cytometry are much higher than those by microscopy [22], suggesting that only the brightest cells are detected by the latter. When TUNEL is used for analyzing native semen samples in flow cytometry, it is important to couple the labelling of DNA breaks to nuclear staining that allows for the exact identification of the sperm population by excluding semen apoptotic bodies [23]. Indeed, the latter may be present in high amounts in subfertile subjects and provoke a heavy underestimation of sDF if included in the fluorescence analysis [24]. Since TUNEL does not have complete access to the sperm nuclei, a previous treatment with dithiothreitol to decondense the sperm chromatin has been added by some authors [25]. However, the hindrance to access into chromatin appears to be present only in non-viable spermatozoa, where chromatin is further condensed with respect to viable ones [26].

Alkaline COMET Assay

In this assay, spermatozoa are embedded in agarose on a microscope slide, where cells are lysed and chromatin is decondensed to remove nucleoproteins and form nucleoids. After unwinding double-stranded DNA in alkaline conditions, electrophoresis provokes the migration of DNA fragments toward the anode and, thus, the formation of a typical comet tail [27] in spermatozoa with DNA fragmentation. Conversely, intact DNA remains in the comet’s head. Then, DNA is stained with a fluorescent dye and, usually, the intensity of fluorescence in the tail is quantified by a dedicated software coupled to a fluorescence microscope (Figure 1C). The COMET assay provides results as mean percentages of tail fluorescence intensity, as scored in 100/200 spermatozoa. However, other COMET output parameters can be considered, including the percentages of comets on the total spermatozoa [13,28,29], thus providing similar results to the other tests. Due to the complete removal of protamines, the COMET assay shows a very high sensitivity. However, the alkaline conditions transform the sperm alkaline–labile sites in DNA breaks, introducing additional damage with respect to the native one [27].

SCD Test

The SCD test is an easy-to-execute assay, requiring only optical microscopy. In this test, spermatozoa are embedded in agarose on a microscopic slide, where cells are denatured with hydrochloric acid and nucleoproteins are removed with a lysing solution. Hence, DNA is stained and slides are observed by microscopy. Spermatozoa with intact DNA exhibit a halo around the central nuclear core, whereas spermatozoa with fragmented DNA do not produce any halo or produce halos with small size (Figure 1D). The sperm tail remains visible, thus increasing the specificity of the test. The molecular mechanism responsible for the formation of the two patterns (presence/absence of halos) is not clear [30]. However, in the original paper, it was shown that spermatozoa without a halo also exhibited the labelling due to DNA breakage detection–fluorescence in situ hybridization, an alternative procedure to detect DNA breaks [30]. An obstacle with this test is the subjectivity by which the size of the halo is established. The criteria to classify sperm halos reported by the last edition of the WHO Manual for Semen Analysis [16] can be of help to score sDF. In addition, computer-assisted systems have emerged to automatically measure the size of halos, thus improving the accuracy and reproducibility of sDF determination with this test.

### 2.1. Comparing Tests for sDF Detection

Unfortunately, till now, the gold standard test to detect sDF has not been established, raising the need to compare the available tests. As deduced by the above description, the four tests do not detect the same type of DNA damage. Usually, it is believed that TUNEL and COMET assay reveal the real DNA breakage, whereas the SCSA and SCD tests detect anomalies in the sperm chromatin. From the point of view of reliability, only SCSA relies on a standardised procedure that minimises the inter-laboratories’ variability and provides reliable cut-off values. In principle, for the other tests, each laboratory should build its own cut-off value to discriminate between fertile and infertile men. Regarding the clinical correlates of these tests, it is obvious that the best test is the one that best predicts the reproductive outcomes. The ability to distinguish infertile men from fertile ones is present with all tests, but TUNEL and COMET yield the best results in terms of the prediction power of natural conception [12]. TUNEL and COMET also appear to be the better tests for detecting the impact of sperm DNA breakage on the pregnancy rate of couples treated by IVF/ICSI [31,32]. However, the association between the amounts of sDF and the outcomes of assisted reproductive technologies (ARTs) remains weak and conflicting [33,34].

In routine clinical practice, ease of execution and scarce requirement of equipment make the SCD test one of the most affordable tests. Conversely, the need for flow cytometry limits the number of clinical laboratories that can implement tests like SCSA and flow-cytometric TUNEL. TUNEL in fluorescence microscopy and the alkaline COMET assay require a mild commitment, although the latter takes a long time and it is difficult to standardise, even in the same laboratory.

The presence of so many different tests for sDF detection generates confusion, especially in clinical practice. An effort to indicate the most suitable test is necessary, with studies where the available tests are simultaneously used for sDF detection and compared in terms of the prediction of reproductive outcomes.

### 2.2. Novel Tests to Detect sDF

All the described tests are unable to differentiate between single- (SSBs) and double-stranded DNA breaks (DSBs). DSBs are believed to be a more severe DNA damage than SSBs because they are more difficult to repair and prone to mis-repair. In somatic cells, DSBs are responsible for the production of chromosome aberrations [35], whereas in spermatozoa, high levels of DSBs have been associated with an increased risk of miscarriage [36,37]. Traditionally, DSBs are detected by the neutral version of the COMET assay, which uses non-denaturing conditions and where the removal of nucleoproteins and the electric field push double-stranded fragments to move away from the nucleoid and form the comet tail [38].

Recently, two novel tests that are able to detect DSBs have been published, namely the SDF-DSBs assay [39] and the LensHooke^®^ R11 [40]. The two tests are similar modifications to the SCD test, where the step of DNA denaturation is omitted. Thus, only double-stranded fragments diffuse in the gel forming a halo. The SDF-DSBs assay uses agarose for the gel and distinguishes cells with and without DSBs by the size of the halos (larger when DSBs are present) [39]. LensHooke^®^ R11 [40] uses polyacrylamide for the gel and distinguishes between the presence (cells with DSBs) and the absence (cells without DSBs) of halos [40]. Future clinical studies will tell whether the detection of only DSBs with these two novel tests will be able to add data on the impact of sDF on miscarriage risk and improve the diagnosis of recurrent pregnancy loss. If so, ease to execute, time-saving, and poor request of equipment make these tests particularly suitable for routine clinical practice.

## 3. Mechanisms Inducing sDF

One relevant point for clinicians is how to treat men with high levels of sDF. In addition to counselling on lifestyle factors impacting sDF [41], the possible treatments for decreasing sDF levels strictly depend on the cell mechanisms responsible for the generation of sperm DNA breaks. Three main mechanisms have been well-established, two necessarily acting in the testis (i.e., abortive apoptosis and defects in sperm chromatin maturation) and one that can act also after spermiation, during the transit in the male genital tract and after ejaculation (i.e., oxidative attack). It has also been hypothesised that a fourth mechanism acts in the epididymis and vas deferens. Here, mature spermatozoa would break their DNA-involving nucleases sequestered in epydydosome-like structures as a part of an apoptosis-like process [42].

Abortive apoptosis refers to the fact that cells programmed to die are not eliminated by local phagocytosis, and indeed, variable amounts of apoptotic bodies are found in the semen of subfertile subjects [24] altogether with apoptosis-like signs in ejaculated spermatozoa [43,44,45,46]. Testis apoptosis is induced by several stimuli [47], and it has also been proposed that it would be triggered at those spermatogenetic stages where the apoptotic machinery, similar to the somatic one, is still present in germ cells [48].

The other testicular mechanism generating sDF occurs during nuclear remodelling in spermatids, when protamines substitute histones. Here, in order to promote nucleoprotein replacement, topoisomerases cut the DNA, producing physiological breaks that are later repaired [49]. However, an impairment in this process could provoke the maintenance of DNA breaks up to mature spermatozoa. For instance, we can speculate that cells committed to death fail to complete chromatin maturation and, in particular, to re-ligate DNA breaks, as a huge overlap between caspase activity and chromatin immaturity has been reported [50].

Oxidative attack is the result of an excess of reactive oxygen species (ROS), overwhelming the antioxidant defences and provoking damage to macromolecules, including DNA. Oxidative attack is believed to be responsible for the increase of sDF in ejaculated spermatozoa when compared to the testicular ones [51], although the involvement of epididymal nucleases has also been proposed, as mentioned [36]. Oxidative stress also causes DNA damage during in vitro sperm manipulation, such as cryopreservation [52], selection [53], and incubation [54]. In mature spermatozoa, if ROS act by directly breaking the phosphodiester backbone of DNA or triggering an apoptotic program, this is not yet clear. ROS are small molecules that can easily penetrate the membranes, reach the nucleus, and break DNA. Conversely, the action of apoptotic nucleases would be hindered by the highly compacted sperm chromatin [55], at least in the toroid structures [36]. A role in generating sperm DNA breaks in mature spermatozoa has also been recently proposed for topoisomerases, as they persist in defective spermatozoa and could be activated by oxidative stress signals [56].

### 3.1. Contribution of Each Mechanism

**Figure 2:**
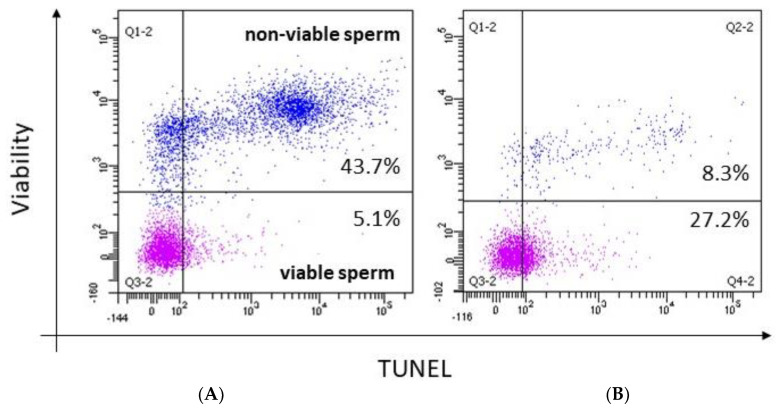
SDF detected by TUNEL in viable and non-viable spermatozoa of a native semen sample (**A**) and after selection (**B**). Note that sDF in the native semen sample is mainly associated with non-viable spermatozoa. Note also that selection deletes a large part of non-viable DNA fragmented spermatozoa and can induce de novo sDF in the viable fraction.

**Figure 3:**
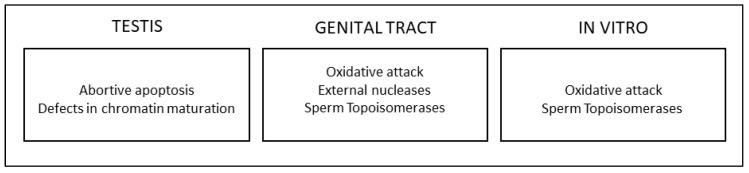
Mechanisms and sites of origin of sDF.

The above mechanisms are not alternative causes of sperm DNA breakage but can occur in the same subject or even concur to generate sDF, posing the point of the contribution of each mechanism in inducing sDF. A study by our group investigated the association between sDF and apoptosis, immaturity, and oxidative damage in the semen of subfertile subjects [24]. We found a different result, depending on whether studying total spermatozoa (viable and non-viable) or only the viable sperm fraction. Indeed, in the total spermatozoa where the main part of sDF is due to non-viable cells ([57,58] and Figure 2), a large overlap between DNA breakage and signs of apoptosis or immaturity was observed. On the contrary, only a few spermatozoa with fragmented DNA also showed oxidative damage to the membrane or DNA [50], a finding recently confirmed by the lack of correlation between the detection of 8-hydroxy-2′-deoxyguanosine and TUNEL [59]. When we analysed the viable sperm fraction, DNA breakage and oxidative DNA damage were largely concomitant, suggesting that ROS play a role above all in viable DNA-fragmented cells. We interpreted ejaculated non-viable DNA fragmented spermatozoa as cells where the onset of DNA damage occurred far from ejaculation, likely in the testes. Ejaculated, viable DNA-fragmented spermatozoa were considered as cells where DNA fragmentation started recently, likely during the transit in the male genital tract [50]. According to this model [50], the bulk of sDF, where the main part of DNA breakage is associated with non-viable spermatozoa, is caused by an impairment of testicular processes, i.e., apoptosis and chromatin maturation. Conversely, oxidative attack would act after spermiation, particularly in those cells that have not yet completed epididymal chromatin compaction [50]. This view is consistent with the fact that only in non-viable spermatozoa does sDF does correlate with the quality of the spermatogenetic process, whereas in the viable ones, such correlation disappears [58], suggesting that the origin of sDF in viable spermatozoa is located after spermiation. As mentioned, the lack of overlap between sDF and oxidative damage in native semen samples was observed in subfertile men [51], and it cannot be ruled out that, in particular conditions of local or systemic oxidative stress, ROS play a role in inducing testis apoptosis, as suggested by studies on animal models [60,61]. It is also possible that ROS act as triggers of the testicular apoptotic program, without a direct role in damaging germ cell structures due to the presence of effective testicular antioxidant defences. This view is also consistent with the lack of oxidative damage in the main part of native sDF [50,59]. (Figure 3).

### 3.2. Treatment Strategies for High Levels of sDF

It was expected that the treatments for high levels of sDF, consisting of the above mechanisms inducing sperm DNA breakage, should be effective. Conversely, we are still far from a general consensus on their use in clinical practice. Treatment with FSH, a possible factor inhibiting testis apoptosis [24,62,63] and promoting sperm maturation [64,65], has shown a beneficial effect on sDF levels in several studies [12]. However, definitive conclusions about the effectiveness of the hormone have not been reached, as it has not been fully studied as to whether the benefit for sDF levels translates into improvements in reproductive outcomes [66] The oral antioxidants have been extensively investigated in the last decades, and many studies reported on their ability to ameliorate conventional and advanced semen parameters, including sperm DNA integrity (the reader is referred to two recent reviews [67,68] for updated results). However, the literature on oral antioxidants is very heterogeneous for the type of administrated compound, dose, and time of treatment and usually neglects the presence of high levels of sDF among the inclusion criteria for patient recruitment. Maybe due to these limitations, in 2019, a Cochrane meta-analysis failed to reveal a benefit of antioxidants for men with high levels of sDF [69]. On the other hand, the current evidence is inconclusive regarding the impact of oral antioxidants on pregnancy and live-birth rate [70]. A third approach consists of recovering spermatozoa from the testis and using them for ICSI instead of the ejaculated ones. Indeed, it has been reported that levels of sDF are lower in testicular versus ejaculated spermatozoa [71] and that this strategy can improve the pregnancy rate, miscarriage rate, and live-birth rate [71,72]. However, a recent meta-analysis concluded that this approach cannot still be recommended for routine clinical practice, due to the low availability and quality of evidence of studies so far published [73].

Overall, it appears to be necessary to further investigate the origin of sperm DNA breaks and, thus, the possible treatments for reducing sDF.

## 4. The Biological Meaning of Sperm DNA Breakage

The mechanisms by which sperm DNA breaks impact reproduction remain elusive. A late paternal effect acting at the eight-cell stage of the embryo, when paternal genes are activated, has been proposed [74,75], and the importance of oocyte repair of DNA damage brought by the male gamete has been demonstrated [74].

In addition, according to some authors, it would be the presence of DSBs that delays embryo development and eventually provokes implantation failure with mechanisms similar to those inducing miscarriage [76].

The impact of sDF might be extended behind the actual DNA breaks, as detected by the available tests, according to the so-called “tip of iceberg theory” [77]. Such theory was first proposed by Evenson in the early 2000s to explain why sDF percentages higher than the 30% threshold blunted natural conception, albeit with the remaining 70% of spermatozoa with apparently intact DNA [77].

This theory, indeed, proposed that the detected sDF might be only the visible part of a more extended abnormality in the sperm population. The same theory could explain why, surprisingly, DNA fragmentation in non-viable spermatozoa (non-viable sDF) predicts natural conception similarly to DNA fragmentation in viable spermatozoa, despite that the former should not have the ability to reach the oocyte and, thus, to impact reproduction [50]. Again, non-viable sDF might be the index of a larger, non-detected abnormality of the sperm population somehow impacting reproduction. It has also to be considered that most spermatozoa with DNA fragmentation in native semen samples are non-viable [43,51] (Figure 2), thus non-viable sDF is similar to the total (viable + non-viable) one. According to the “tip of iceberg” theory, it can be hypothesised that only when sDF exceeds a certain threshold, the impairment of the sperm’s function/structure is extended to the whole sperm population, highly reducing the probability of natural pregnancy. However, lower values of sDF would not guarantee a successful task of spermatozoa. Indeed, although it is possible that a fraction of the spermatozoa is free from defects related to DNA breakage when the sDF level is low, other types of sperm impairments may hinder reproduction.

Cells with a hidden abnormality related to sDF are likely those developing an evident DNA breakage when ejaculated spermatozoa are submitted to an insult, for instance in vitro incubation or sperm selection. The dynamics of sDF during in vitro incubation have been extensively studied [78,79,80]. In these conditions, one could expect that all subjects undergo similar damage. Conversely, the amount of the developed damage is highly variable among individuals and much more variable than the basal value of sDF (i.e., before incubation) [81] and might depend on the variable extension of a vulnerability due to the above hidden abnormality. Some authors called the amount of DNA fragmentation developed during incubation “cryptic” or “latent” sperm damage [79], which is a similar way to interpret the cause of de novo induced DNA damage. A similar situation is observed in de novo sDF developed during swim-up or density gradient centrifugation (DGC), where the amount of the induced sDF is highly variable among subjects as well [82]. For instance, after DGC, in addition to subjects who, as expected, decreased sDF during selection, a relevant fraction of subjects increased DNA breakage, in some cases, up to 100% [83]. Again, we can hypothesise that subjects increasing sDF with selection have a cell vulnerability to the induced damage that is not present in the subjects where selection decreases sDF.

The nature of this vulnerability/abnormality is not clear. Interestingly, Gosalvez et al. [79] reported a direct correlation between the amount of sDF developed during in vitro incubation and the basal P1/P2 ratio [82]. This finding suggests a role for poor chromatin structure due to impaired maturation. Also, the presence of scarce antioxidant defences, the persistence of topoisomerases [84], or a variable combination of these conditions is reasonable. Given the possible impact on reproduction, future studies should investigate these hidden aspects related to sDF in sperm populations.

## 5. The Sperm Population Where sDF Should Be Detected

As mentioned, for predicting natural conception, the small sperm population reaching the oocyte appears, in principle, the most suitable target. However, the experience with sDF showed that the detection of just one parameter in the bulk of native semen samples can discriminate between fertile and infertile men [11,12]. As discussed above, the “tip of iceberg theory” hypothesises that this is possible, as sDF signals overall damage to the sperm population.

**Figure 4:**
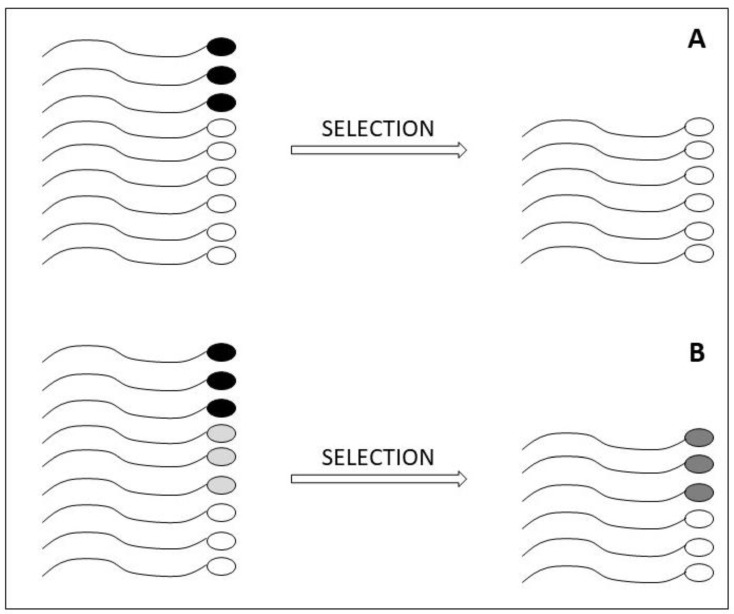
Two hypothetical changes in sDF amounts during sperm selection, starting from similar sDF amounts. In (**A**), selection deletes non-viable DNA fragmented spermatozoa and, thus, reduces the percentage of sDF. In (**B**), selection deletes non-viable DNA fragmented spermatozoa but induces a de novo damage in viable spermatozoa, thus increasing the percentage of sDF. Induction of sDF is due to a non-detectable sperm abnormality, present only in (**A**). Black heads, non-viable DNA fragmented spermatozoa; white heads, healthy spermatozoa; pale-grey heads, spermatozoa with a hidden abnormality; dark grey, viable DNA fragmented spermatozoa.

In assisted reproduction, the tip of iceberg theory might also explain why several studies found an impact on reproductive outcomes by sDF detected in a native semen sample [25], whereas, in principle, the sperm population for sDF testing should be that one selected for oocyte insemination. The relationship between the amount of native sDF and that one in the selected spermatozoa is complex and dependent on the individual. In clinical practice, sperm preparation is mainly conducted by selection with DGC or swim-up procedures. During such procedures, most non-viable spermatozoa, which are mainly DNA fragmented, are deleted, thus decreasing sDF in the selected population. Conversely, viable spermatozoa may both decrease sDF and, in a relevant fraction of patients, undergo a de novo induction of sDF [65] (Figure 2), possibly deriving from hidden abnormalities of spermatozoa, as discussed above. Hence, in a native semen sample, most of the sDF is due to non-viable spermatozoa, whereas in the selected population there is the opposite situation. Indeed, when sDF is relevant, it is mainly associated with highly motile spermatozoa [12,65]. In addition, the amount of sDF in the selected spermatozoa is not predictable by the amount of sDF before selection. Indeed, selected samples with high sDF (due to an increase of DNA breakage during selection) show similar basal, i.e., before selection, values of sDF as selected samples with low sDF (due to a decrease of DNA breakage during selection) [12]. Importantly, subjects increasing sDF during selection subsequently show a 50% lower pregnancy with respect to those decreasing the damage [12]. Hence, it is possible that very different amounts of sDF in the populations used to inseminate the oocyte correspond to similar values of sDF in the native semen sample (Figure 4). This aspect could represent one factor weakening the impact of native sDF (used by many studies, [25]) on the outcomes of assisted reproduction, in addition to those due to possible bias in the recruitment of couples [56,85]. Unfortunately, very few studies compared sDF in native and selected spermatozoa in terms of impact on ARTs, and the results are contradictory and apparently dependent upon the test used to reveal DNA damage [82,86,87]. These studies could suggest whether the detection of sDF in a selected sperm population might better predict the reproductive outcomes in couples treated by ARTs, and which is the best test to be used.

It has also to be considered that alternative procedures to select spermatozoa for oocyte insemination have been emerging [88,89]. In particular, microfluidic technologies promise to select spermatozoa based on not only motility but also properties such as chemotaxis and rheotaxis [90]. Future studies will tell us whether these novel tools are able to completely avoid DNA defects in selected spermatozoa and to improve ART outcomes.

## 6. Conclusive Remarks

Many laboratories added tests to detect sDF as an adjunct to routine semen analysis. However, some points related to sDF are still open.

The available tests to detect sDF are different each from other and likely do not reveal the same type of damage. In addition, for tests lacking a standardised procedure, it is not reliable to use the cut-off values established in other laboratories. Given the confusion generated by the existence of so many different tests, studies aimed at establishing a gold standard test are necessary.

Mechanisms originating sDF have been extensively studied and prompted several treatments for high levels of sDF. However, consent on the clinical use of such treatments has not been reached so far. Hence, further research is requested on mechanisms responsible for sDF and suitable treatments for men with high values of this sperm damage.

The existence of hidden sperm abnormalities linked to sDF deserves novel studies. Hopefully, these studies could help to understand the impact of sDF on reproductive outcomes.

Finally, studies assessing sDF before and after sperm preparation could help to better understand the impact of sperm DNA breakage on ART outcomes.

## Figures and Tables

**Table 1:** Key features and main advantages/disadvantages of the tests for sDF detection.

		SCSA	TUNEL Fluorescence Microscopy	TUNEL Flow Cytometry	Alkaline COMET Assay	SCD Test
Key features		Reveals the susceptibility of sperm DNA to denaturation. Flow cytometry	Reveals DNA breakage by enzymatically labelling the 3′OH ends of DNA	Reveals DNA breakage by enzymatically labelling the 3′OH ends of DNA	Reveals DNA breakage by electrophoretic migration of DNA fragments after sperm lysis/decondensation and DNA unwinding. Fluorescence microscopy	Reveals the ability/inability to disperse DNA fragments after sperm lysis/denaturation. Light or fluorescent microscopy
Advantages	Use of very low sperm number (thousands)	✓	✓		✓	✓
	Use of frozen semen samples (or dry specimens)	✓	✓		✓	✓
	Presence of a standardised procedure	✓				
	Analysis of thousands of spermatozoa	✓		✓		
	Coupling to detection of other parameters			✓		
Disadvantages	Request of experts in flow cytometry	✓		✓		
	Request of a specific software	✓			✓	
	Request of a high number of spermatozoa (millions)			✓		
	Labour intensive				✓	

✓, yes.

## Data Availability

No new data were created or analysed in this study. Data sharing is not applicable to this article.
